# Monthly Summary of Medical Journalism

**Published:** 1860-06

**Authors:** O. C. Gibbs

**Affiliations:** Frewsburg, N. Y.


### ECLECTIC DEPARTMENT,

### AND SPIRIT OF THE MEDICAL PERIODICAL PRESS.

*Monthly Summary of Cotemporary Medical Journalism.* By O. C. Gibbs, M.D., Frewsburg, N. Y.

*Reduction of Strangulated Hernia.*—In the *Maryland and Virginia Medical Journal* for April, Dr. Stockdell reports a case of strangulated congenital hernia in a child three months old, that was reduced without an operation, in the treatment of which there are some points of interest worthy of making a note of. *Taxis* was tried "under the full influence of an anodyne enema followed by a warm bath;" also, subsequently, "under the influence of an *anæsthetic*," but entirely without favorable results. "A tobacco leaf was procured and thoroughly moistened with warm water, applied to the whole abdominal parietes. At 1 o'clock, its effects being only slightly perceptible in the pulse—the abdominal distention remaining unchanged—it was removed and flannel cloths wrung out of a very strong, hot infusion of tobacco, directed to be applied in its stead and frequently changed." The tobacco was continued from nine in the morning until seven in the evening, when the hernia was spontaneously reduced.

*Ovariotomy.*—In the April number of the *Md. and Va. Medical Journal*, Dr. B. Roemer, of Otter Bridge, Va., reports a successful case of ovariotomy. We intend that our *Summary* shall contain a record of all such successful operations, performed in this country, the report of which comes to our eye.

Dr. Roemer's patient was 38 years old, and the mother of eight children. The tumor was of three and a half years' growth, and, when removed, it weighed, with contents, 25 pounds. The patient made a good recovery, no symptoms of inflammation having manifested themselves.

*Bloodletting in Inflammation.*—In the *Lancet and Observer* for April, Dr. J. F. Hibbard, of Richmond, Ind., has a lengthy article upon general bloodletting in the treatment of inflammation. In our *Summary* for April we gave a brief synopsis of a paper upon the same subject by Prof. L. M. Lawson. Dr. Hibbard supports the opinions of Prof. Bennett, while he takes issue with the views and reviews the arguments of Prof. Lawson. Dr. Hibbard believes, with Professor Bennett, that inflammation is the same now that it has ever been, and that the change in treatment is not so much the result of a change in the type or grade of inflammatory action, as it is the result of a change in the views and opinions of practitioners. Though we should be glad to analyze the paper before us, we must be content with a few quotations. Dr. Hibbard says, "The purpose and effect of general bleeding is to reduce vital action and lessen nutrition; but as the inflammatory fever is the evidence of an already existing depression in vital action and lessened nutrition, and is the thing to be removed, we cannot apply bleeding for this purpose until we are ready to adopt the doctrine that to cure a disease we must amplify its pathology, which would be absurd. And thus we determine a negative answer to the query, whether bleeding can abate or control inflammatory fever in a manner that tends to a restoration of normality. It has already been shown that inflammation, as a local disease, cannot be benefited by general bloodletting. We now arrive at the conclusion that in inflammatory fever it is equally impotent for good, and hence, led by our course and method of study, we are prepared to answer the question, Can general bleeding prevent or arrest inflammation? It cannot.

Theory, therefore, in her own pleasant way, steps in and lends the shoulder of science to support the world-wide practice of the abandonment of general bleeding for the purpose of cutting short or arresting inflammation. And this she does, not by soothing us with the assurance that years ago inflammation was a great behemoth, and we did right to jugulate him, while now it has become a caged animal that we must feed and cultivate to its destiny, but by telling us distinctly that it is the same to-day that it ever was, and that now we have light and see our past errors; we should be true to our manhood and adorn our profession, by being willing on all suitable occasions to acknowledge that we were once in the dark, and honestly did wrong because of that darkness."

*Yellow Fever—New Treatment for.*—Prof. E. D. Fenner, Editor of the *New Orleans Medical News and Hospital Gazette*, in the fall of 1858 called attention to the use of *veratrum viride and chlorine* in yellow fever. In the spring of 1859 he published full directions for their use. Prof. Fenner's experience was based upon more than 50 cases treated with unusually satisfactory results.

In the same journal for April, Dr. William McCraven, of Houston, Texas, has an article upon the treatment of yellow fever. Dr. McCraven adopted in part the suggestions of Prof. Fenner, and, like him, reports quite satisfactory results. Dr. McCraven usually emptied the stomach in the first place with mustard, or simple warm water; this was followed by a mercurial purge, and when the fever was fully developed he cupped the head, stomach, or back, and commenced immediately with the following mixture:

"℞.—Tincture of veratrum viride, ʒj.

Tincture of aconite, ʒj.

Sulphate of morphia, gr. j.

Syrup of orange peel, ℥iv.

Water to make a four-ounce mixture. Give a teaspoonful every two hours during fever. This was usually continued for thirty-six or forty-eight hours." . . . . " After adopting the course outlined, I have never been more successful. I was at one time in the midst of the epidemic twenty-five days without the loss of a single patient, and I lost but two under this course of treatment. My patients were much relieved, unquestionably, by the emptying, and I think the mercurials were clearly indicated by the quantity of bile; but still I feel assured that the veratrum and aconite influenced the results to a considerable extent. I had heretofore been prejudiced against veratrum in yellow fever; I feared its irritating effects on the stomach, but in practice I found exactly the reverse. I think I cannot be mistaken in the notion that by its influence on the circulation it prevents in a great measure those congestions, both of stomach and kidneys, which terminate so often and so fatally in black vomit and suppression of urine."

*New Treatment for Congestive Chills.*—In the *N. O. Medical News and Hospital Gazette* for April, Dr. J. E. Keator reports a case of congestive chill, of almost hopeless character, which was promptly relieved by the internal administration of chloroform. He says, " I commenced by giving five drops in a little water. Within less than two minutes after the dose was swallowed the girl remarked that the burning at her stomach was gone, the vomiting ceased, and she seemed to rest a little. I continued giving it at intervals of from ten to fifteen minutes, for nearly two hours, in the same quantity as at first, and each time had the satisfaction of hearing from the lips of the patient herself, that it made her stomach feel so much better that she wanted more of it. At the end of two hours the pulse was full and strong at the wrist, the extremities were warm, and reaction was fully established." Quinine, in full doses, was next administered, another chill prevented, and the patient soon restored to health.

*Cause of Goitre.*—In the *Louisville Medical News* for March is published an inaugural essay by Dr. N. W. Brown, of Mexico, upon the subject of goitre. It would seem, from the Doctor's article, that this affliction is a very common disease in many parts of Mexico. After eight years of observation in the goitrous districts of Northern Mexico, he concludes that the disease " is produced by the use of water impregnated with minerals." These minerals are "iron, copper, lead, sulphur, and alum, in their many different forms and combinations existing in nature."

Dr. Brown says we seldom find minerals in Mexico at an altitude above two or three thousand feet, and that up the mountains, above the mineral line goitre does not occur. He says, further, that he never knew goitre to occur endemial "where the inhabitants thus effected did not use water impregnated with *minerals*, quite perceptible even to the taste."

*A New Remedy for Consumption.*—In the *Louisville Medical News* for March, a writer that we strongly suspect may be Dr. J. Lawrence Smith, in a review of *Headland on the Action of Medicines*, incidentally refers to a new remedy for tuberculosis. The remedy is a combination of fresh *linseed oil and whiskey.* Of it the writer says, "In a reasonable experience of five or six years the results are so encouraging that we have no disposition whatever to fall back upon the more offensive and very expensive article which is said to be obtained from the liver of the cod-fish." . . . . "In the use of the linseed oil and whiskey, we are in the habit of ordering a teaspoonful of each three times a day, and as the stomach becomes accustomed to it, increasing the quantity to a tablespoonful. When they are put in the same bottle and shaken up thoroughly, before use, the taste of the oil is in a great measure covered up by the spirit, and is very rarely offensive even to delicate stomachs." . . . . "It would be mere repetition to go over the words cough, expectoration, hectic, emaciation, night-sweats; but all these things have we seen pass away under the use of the oil and whiskey, and a few months have brought up the patient to normal weight, normal appearance, and normal sensation. It is not designed to claim for the linseed oil any medicinal advantages over the cod-liver; but if it is more certainly pure, if it is cheaper, if it is less offensive, and accomplishes what is claimed for the fish oil, it certainly commends itself as a boon to poverty and to sensitive stomachs, and as such we commend it to the profession."

We think this combination would not be amenable to one of the objections urged by Dr. Bull against whiskey alone—that of inducing intemperate habits!

*Inversion of the Uterus.*—In the April number of the *Cleveland Medical Gazette*, is published the first part of the deposition of our esteemed friend and preceptor, Prof. John Delamater, in the Fisher case, now pending in the courts of Chicago. In the paper before us, Prof. Delamater gives expression to some opinions in regard to inversion of the uterus that are not in accordance with the traditional opin ions of the profession. It is the opinion of the authorities in this matter, that inversion commences by depression of the fundus of the uterus; which depression must have its origin in the last labor. Prof. Delamater thinks that inversion may, in some cases, commence at the neck of the uterus. After describing the manner of inversion by depression of the fundus, he says, " But there are a few cases in which the womb is very differently situated, especially after severe labors, in which the powers of the womb, as well as of the general system, have become exhausted. Frequently such a loss of power and action on the part of the womb, arises from extreme loss of blood, either during the labor, or after delivery, sometimes, notwithstanding that the womb is known to have contracted favorably at the close of the labor; yet falling subsequently into a state of relaxation, extreme bleedings have ensued, and continued until the womb has become as soft and pliable as a wet ox-bladder, while from the entire relaxation, the flow has passed away so freely as to allow of no accumulation within to preserve its cavity and prevent a collapse of its inner surfaces upon one another, insomuch that there may remain no cavity into which the flabby fundus could be depressed. It is evident, as I apprehénd, that the process of inversion in such a case must be different from the common opinion of it. I have several times passed my hand into wombs situated precisely as last above described, for the purpose of exciting contraction by the stimulus of the moving hand within it; and sometimes have in that way, also, introduced special agents for rousing it to activity, and revolving such. Cases in my practice have occurred where the smallest special pressure upon it could hardly fail to force it down through its mouth, equally relaxed and void of all resistance, as I believe to have occasionally happened. I have said to myself, it is impossible that in such a case, the inversion should begin by depression of the fundus into the cavity of the organ; but on the contrary, that subject to pressure tending to force it downward, in such a case, it would inevitably be crushed into an irregularly-folded mass, which must emerge from its narrower mouth in an order commencing immediately above the neck. If the mouth were extremely yielding, and the impulsive force adequate, the whole of the organ would be forced through so suddenly as to render it impossible to trace the order of its emergence by the touch, even though the finger were at the moment within the vagina."

*Malarial Fever.*—That intermittent fever is in some way dependent upon heat, moisture, and vegetable decomposition, for its causatives, has long been the commonly received doctrine among medical teachers. It has been supposed that vegetable matter was all-important, heat and moisture being only necessary to bring about the necessary decomposition to develop and render gaseous the requisite poison. Now and then, a dissenting voice has been raised, and new theories propounded, but, so far, without success in gaining anything like general assent among intelligent members of the profession. It is true, unanswerable objections have been raised to the vegetable origin of the malaria, but each new system has been subject to as many, if not more, equally fatal objections, and the profession, for want of a better, still clung to the old theory.

In the *Nashville Journal of Medicine and Surgery* for January and April, its editor, Prof. W. K. Bowling, has articles upon the subject of malarial fever, in which he gives expression to his views, which, differing from the old theories in regard to the causatives of this disease, are of interest to those who have seen objections to the vegetable miasm theory. He says: "It has been shown again and again, by as truthful and competent observers as ever lived, that every variety of malarial disease exists where no vegetation exists or can exist. And in every such locality, without a single exception, it is in proof that water existed in such a relation to intense solar heat, as to prevent rapid evaporation. What then, if we do find water in the same relation to solar heat in other localities, with decomposing vegetable matter present, when it is demonstrated, that whether it is present or absent, the result is precisely the same.

"Again, it has been repeatedly shown by observers equally as truthful and competent, that vegetation in vast quantities may rot, and fill the air for miles around with its vile stench, where water does *not* sustain the proposed relation to solar heat *without* producing malarial disease, or any sort of disease. Are we to throw aside all these suggestive and precious observations for the poor parrot privilege of repeating forever the words of the great *auctor princeps* upon paludal emanations?"

Prof. Bowling's opinions in regard to the development of malaria are thus enunciated: "A certain relation of watery fluid to long-continued and intense solar heat, always secures malaria, *and nothing else will*, is my own doctrine; and I believe, the only one that will account philosophically for all the facts which we are compelled to admit in connection with the etiology of malarial fever. This relation of watery fluid and solar heat must be such, as that the latter can operate powerfully upon the former, while the former is so situated as to prevent rapid evaporation."

*Trismus Nascentium treated with Quinine.*—In the *Chicago Medical Examiner* for March, Dr. H. A. Johnson reports a case of trismus nascentium cured by the administration of quinine. Deriving no benefit from previous treatment, he says: "At 11 o'clock in the evening of the second day, thirty-four hours from the first attack, I gave the little patient one grain of sulphate of quinine. In a few minutes a severe spasm came on, differing in no respect from the fifty or sixty that had preceded it. The next interval was a full hour, a longer period than any which had hitherto occurred. The paroxysm was slight and shorter in duration than usual. For the next two hours the patient rested in a sweet and gentle sleep. On awakening, there was a little closure of the jaws, and slight spasm of the muscles of respiration, but nothing in comparison to the fearful contortions that we had previously witnessed. For the next five hours there was not the first symptom of a convulsion, but at 7 A. M. the lips became somewhat livid, and the breathing irregular. This lasted only for a few minutes. I immediately gave another grain of the sulphate of quinine. From that time there has been no symptoms of a return of the disease, and the child is now perfectly healthy." This is Dr. Johnson's second case of cure with quinine, and, if we rightly remember, the same treatment has previously been successfully resorted to in one or more cases.

*Treatment of Croup.*—Before the Chicago Academy of Medicine, as per report in the *Medical Examiner* for March, Dr. Davis approved the use of the *Turpeth mineral* in croup, which had proved very successful in his hands during a period of nine years. He said "he was in the habit of urging its claims in his lectures to his class, and he relied mainly upon it in the treatment of this disease."

*Subcutaneous Injection.*—In the several issues of the *Boston Medical and Surgical Journal* for April, Dr. A. Ruppaner has a serial article upon the treatment of neuralgia by the subcutaneous injection of narcotics, &c., illustrated with cases. Dr. Ruppaner holds the following language: "In all cases where the cutaneous, and particularly superficial cutaneous nerves have been the seat of the malady, this treatment has answered my most sanguine hopes. Even in cases of long standing, when combined with appropriate constitutional treatment, I have succeeded in giving relief, for a considerable period of time, to the painfully harassed patient, after all other possible expedients had been tried in vain.

"And let me here append a few words in regard to constitutional treatment in neuralgia, as one of the conditions of success. In almost every case that has come under my observation, a tonic treament has been indicated. I have tried both mineral and vegetable tonics, and must give the preference to vegetable tonics. I have used the sulphate of quinine in many cases, and in all but one it was followed by good results. I am of opinion that a tonic treatment ought at once to be adopted, with few exceptions; and that the same ought to go hand in hand with the local treatment."

*Vomiting in Pregnancy.*—Before the Louisville College of Physicians and Surgeons, as per proceedings in the *Medical News* for April, Dr. Thum said "he had found bi-sulphate of soda and crab cider a specific for vomiting of pregnancy." Another member present, whose name is not mentioned, stated that he had a case of very distressing vomiting associated with pregnancy, under his charge, and after failure of all his prescriptions, had advised cold hip baths. The husband had modified this suggestion by pouring a pitcher of cold water down the spine, with prompt alleviation of symptoms whenever resorted to. Several members had used the *oxalite of cerium* with quite satisfactory results in similar cases, and others entirely without benefit.

*Hypophosphites in Spurious Hydrocephalus.*—In the *Medical Press* for March 31st, we made some remarks upon the use of the syrup of the hypophosphites, in some conditions of the brain in young children, with an illustrative case. That the brain and spinal marrow contain phosphorus largely, is well known, and it is quite probable that deaths occasionally occur from nervous prostration or exhaustion of the nervous centres that might be saved by the timely use of the agent under consideration. We have often thought that the use of strychnine was much neglected in conditions as above, for we know we have seen it operate more satisfactorily than any other known remedy, in the advanced stages of typhoid conditions, and we now take pleasure in calling the attention of the profession to an agent against which there is less popular prejudice, and in the the use of which, there is less danger from careless administration, and which may yet be found to fulfill an indication of no small importance.

*Sore Nipples.*—In the *Medical Press* for March 31st, Dr. J. B. Amiss, of Va., gives a new cure for that distressing complaint. He says he has employed many times, and with uniform success, the following: "One part tincture of iodine to three of water." . . . . "The iodine wash was applied with a camel's hair pencil, morning and night, *a few days*, when I had the satisfaction of seeing my efforts to relieve my patients eminently successful." We hope that, in other hands, the remedy proposed by Dr. Amiss may prove equally successful; if so, it will prove a great boon to that interesting and worthy portion of the human race whose duty and pleasure it is to bear and nurse children.

*Strangulated Hernia treated with Opium.*—In the *Chicago Medical Journal* for April, Dr. S. W. Noble has an interesting article upon the above subject. He says: "The treatment that we propose for the relief of strangulated hernia is full and oft repeated doses of opium until complete narcosis is produced. When given in this way, opium will evidently produce a more complete relaxation of the muscular system than any other known remedy. It possesses this advantage over most other relaxing medicines—you can manipulate without giving pain. But on the other hand, if you relax the system perfectly with tartar-emetic or tobacco, and apply the taxis, you cause pain in the parts which excite contraction in the muscles implicated in the hernia, which are the ones you wish to relax. The same objection applies to chloroform, with this slight exception—the patient is not conscious of the pain, and you have not the influence of volition to contend with.

Opium, likewise, when given in large doses, say from three to five grains to an adult, arrests vomiting, which is one of the most unpleasant symptoms, quiets the irritability of the system, and effectually prevents inflammation and mortification in the strangulated portion of the bowel. When the patient is thoroughly and permanently under the influence of opium, if the stricture is not relieved and the bowel returned spontaneously, it may be readily accomplished by a renewal of the taxis. At least, that has been my experience in these cases."

Dr. Noble reports *seven* cases in which the opium treatment has proved perfectly successful, and these, he says, are the only cases of strangulated hernia that have occurred in his practice. On the recommendation of Dr. Noble, Dr. A. H. Luce, of Bloomington, has treated two cases with opium, with success, and Drs. Harrison Noble, of Keyworth, and J. W. Coleman, of Le Roy, have each treated one in the same manner, and with the same results. Thus, *eleven* cases have been reported, with results quite satisfactory. This is certainly an interesting experience, and we have no doubt the suggestion of Dr. Noble will be soon put to a more extended trial.

*Stomatitis Materna.*—In the *Chicago Medical Journal* for April, Dr. L. S. Ellis has an article upon the above subject. The article gives evidence that its author is familiar both with the disease and its literature. We quote his treatment: "I am accustomed to use the two following prescriptions with uniform success:

| | |
|---|---|
| ℞.—Magnes. calc., | ʒj. |
| Sapo. hisp. pulv., | grs. x. |
| Camphor, " | |
| Sang. can., | ää, grs. v. |

Mix. Dose from three to five grains, four times daily.

R.—Cinch. Rub., ℥ss.

Ferri carb.,

Rad. rhei., ää. ʒij.

Port Wine, Oj.

Ft. mist. Dose, table-spoonful with each meal.

When there is loss of appetite, I direct the tonic to be taken half an hour before eating, and the powder soon after; otherwise, both arc to be taken soon after eating. In many cases where there is not much constitutional debility, and in the earlier stages, I find the first prescription amply sufficient to control the disease."

*Arsenic in Menorrhagia, Dysmenorrhœa, &c.*—In the February number of our *Summary*, we referred to a paper by Dr. A. P. Burns upon the use of arsenic in many uterine diseases; the article of Dr. Burns was published in the October issue of the *American Journal of Medical Sciences.* In the March number of the *Belmont Medical Journal*, one of the editors has the following: "Since reading the above article, we have had an opportunity to test the merits of arsenic in several cases of menorrhagia. In all of them it proved successful, without producing any unpleasant effects. We have used it in dysmenorrhœa and leucorrhœa, with results more satisfactory than anything previously used. We suggested its use to a neighboring physician, in a very alarming case of uterine hæmorrhage from placenta previa, some five weeks before accouchement, where there had been previous attacks of a very threatening character, and at all subsequent attacks it controlled hæmorrhage, but always produced a strong and persistent uterine aching; the woman went her full period, and had a speedy and safe delivery under the care of Dr. Campbell, with no unusual hæmorrhage or untoward symptoms."

*Fungoid Growth of the Gums and Jaws.*—In the *Dental Cosmos* for April, Dr. W. D. Holbrook reports two cases of fungous growths from the jaw cured by the application of pulverized charcoal, after a failure of a variety of other means. In the cases reported, the fungus had been removed with a ligature, with the knife, with caustics, and with the actual cautery, all to no purpose, as the disease returned with great rapidity. The application should be made frequently, and the remedy persevered in for several weeks.

*Iodide of Potassium in Neuralgia.*—In the *Maryland and Virginia Medical Journal* for March, (the proprietors will please accept our thanks for the back numbers of this excellent Monthly,) Dr. F. P. Bibby has an article upon the use of iodide of potassium in neuralgia. He says: "I can say, after some experience, that there is no remedy which seems to be more beneficial in neuralgias than the iodide of potassium, in full doses. Exciting the secretions and excretions, it may be supposed to act by eliminating the morbid cause; and in this way, probably, we see its good effect in chronic rheumatism, chronic syphilis, and other analogous conditions, and for the same reason it may be expected to cure neuralgic troubles generally."

*Iodide of Potassium in Diseases of the Brain in Children.*—In the *Maryland and Virginia Medical Journal* for February, is copied an article upon the above subject, from the *Edinburgh Medical Journal*, from the pen of John Coldstream. The editors of the *Md. and Va. Med. Journal* subjoin the following note: "We can fully endorse the benefit of this remedy in tubercular meningitis, if there is any reliance to be based upon a diagnosis made most carefully. During the past year, two cases happened to us in consulting practice, and in neither case was there a difference of opinion with any of the physicians concerning the nature of the disease. This modest but able paper of Dr. Coldstream has forestalled an account of the cases which we had drawn up for publication. But the views here expressed so completely anticipate our own, at the time the paper was written, that we are glad to find a more entertaining substitute by endorsing this."

We are glad to see this able evidence in favor of a plan of treatment we have pursued for years, and also, the still more able endorsement. Mercury and bloodletting, the text-book remedies for hydrocephalus, or rather tubercular meningitis, have done quite too much injury in these cases, and we trust in future reliance may be placed upon safer and more appropriate remedies. In the paper of Dr. Coldstream, surprise is expressed that so few references are on record of the use and utility of this remedy in head affections of children. He makes allusion to such as have fallen under his observation.

We do not complain that he has left our humble name out of the list, but we take pleasure in referring Dr. Coldstream and his endorsers to a paper of ours upon the same subject, published in the January No. of the American Medical Monthly for 1856; also to another in the March No. of the *Medical Examiner* for 1853. In the article first named, we used the following language: "I wish to call more especial attention to the remedial powers of iodide of potassium. In meningeal tuberculosis I believe it to possess an influence second to no other known remedy." . . . "I have used it in several instances with success, in cases that I supposed to be meningeal tuberculosis; cases, too, some of them, in families of which several of the children immediately preceding had died of the disease under consideration, as they respectively became of a certain age, having received the usual antiphlogistic treatment at the hands of eminent and skillful physicians."

For the last eight years, we have used the iodide of potassium in tubercular meningitis, as well as in other head affections of children, and the results have been such as to induce us, on several occasions, to recommend its use to others in similar cases. We will conclude our remarks upon this subject by a quotation from the paper of Dr. Coldstream: "My own experience leads me to regard the iodide as more likely than any other drug to promote this desired end; and my confidence in it, as the remedy best adapted to all stages of tubercular diseases of the head, is so strong, that whatever else might be done or left undone, I would persevere in administering it, even in circumstances the most desperate."

